# Supplementary material for: Engineering substrate specificity of HAD phosphatases and multienzyme systems development for the thermodynamic-driven manufacturing sugars
Source: Nat Commun. 2022 Jun 23;13:3582. doi: 10.1038/s41467-022-31371-8 (PMC9226320; doi:10.1038/s41467-022-31371-8)
Supplement: Supplementary file 1 — Supplementary Information [file 41467_2022_31371_MOESM1_ESM.pdf]

Supplementary Information

Engineering substrate specificity of HAD phosphatases and multienzyme systems development for the thermodynamic-driven manufacturing sugars

Chaoyu Tian[1,2,†], Jiangang Yang[1,2,†*], Cui Liu[1,2†], Peng Chen[1,2], Tong Zhang[1,2], Yan Men[1,2], Hongwu Ma[1,2*], Yuanxia Sun[1,2,*], Yanhe Ma[1,2]

[1]National Engineering Laboratory for Industrial Enzymes, Tianjin Institute of Industrial Biotechnology, Chinese Academy of Sciences, Tianjin 300308, China

[2] National Technology Innovation Center of Synthetic Biology, Tianjin 300308, China

[†] These authors contributed equally to this work

[*] Correspondence: yang_jg1@tib.cas.cn; ma_hw@tib.cas.cn; sun_yx@tib.cas.cn;

Supplementary Note..3

Supplementary Table 1. The enzymatic activity of Pase12 and Pase14 to M6P, F6P, and G6P 4

Supplementary Table 2. The binding energy of mutation sites in cap-domain mutants using Rosetta design method 4

Supplementary Table 3. The enzyme activity of WT and mutants to different substrates...............5

Supplementary Table 4. Properties of enzymes and reactions in multienzyme systems 5

Supplementary Table 5. Design constraints between the substrate phosphoryl group and catalytic residues of Asp8, Ser41 and Lys191...6

Supplementary Figure 1 SDS-PAGE analysis results of 15 candidate phosphatases in this study. 7

Supplementary Figure 2 The $P_{G/F/M}$ values of single-site mutations...8

Supplementary Figure 3 Multi-sequence alignment of Ts38HM6PP with five phosphatases using ClustalW program..9

Supplementary Figure 4 The mannose concentration of different mutations in the screening system.. 10

Supplementary Figure 5 The effect of combinational mutations on $P_{G/F/M}$. 10

Supplementary Figure 6 The Michaelis-Menten kinetic plots for mutants of M6P.......................11

Supplementary Figure 7 The Michaelis-Menten kinetic plots for mutants of F6P. 11

Supplementary Figure 8 The Michaelis-Menten kinetic plots for mutants of G6P........................12

Supplementary Figure 9 HPLC chromatograms to obtain the values of $P_{M/G/F}$. 12

Supplementary Figure 10 The catalytic property of WT, MM3, MF2 and MG7 to different substrates...13

Supplementary Figure 11 SDS-PAGE analysis of used in the biosynthetic systems.....................14

Supplementary Figure 12 The dephosphorylation activity of BaSP to G1P..................................14

Supplementary Figure 13 Standard Gibbs free energy change of each and overall reaction for manufacturing mannose in the biosystem..15

Supplementary Figure 14 Two industrial routes for manufacturing mannitol...............................15

Supplementary Figure 15 The calibration curve for sugars. 16

Supplementary References...17

Supplementary Note

The HPLC system (Agilent 1200) equipped with a refractive index detector (Agilent G1362A) and a cation exchange column (Sugar-Pak^{TM}, 6.5×300 mm) was used for HPLC analysis. Deionized water was used as a mobile phase at a flow rate of 0.4 mL/min, and column temperature was controlled at 80 °C. For post-processing of the data from HPLC analysis, the peak area for target product was obtained by auto and manual integration, and then calculated by the calibration curve for sugars.

Supplementary Table 1. The enzymatic activity of Pase12 and Pase14 to M6P, F6P, and G6P

	M6P (U/mg)	F6P (U/mg)	G6P (U/mg)
Pase12	0.13±0.03	0.18±0.05	0.64±0.08
Pase14	0.02±0.01	0.56±0.11	0.06±0.01

Supplementary Table 2. The binding energy of mutation sites in cap-domain mutants using Rosetta design method

Positions	Top designs		Binding energy (Reu)
Y122	M6P	H	-8.8
	G6P	E	-8.03
	F6P	Q	-10.53
H125	M6P	L	-7.77
		I	-7.49
	G6P	Y	-7
	F6P	D	-9.42
		V	-9.27
L149	G6P	M	-7.36
F178	M6P	V	-8.89
	G6P	L	-7.8
	F6P	H	-10.54
Y181	M6P	F	-8
	G6P	M	-7.68
	F6P	H	-10.4

Supplementary Table 3. The enzyme activity of WT and mutants to different substrates

Substrates	WT	MM3	MF2	MG7
G6P	0.3 ± 0.04	0.5 ± 0.1	0.03± 0.01	2.6 ± 0.2
F6P	0.2 ± 0.05	0.4 ± 0.1	2.9 ± 0.2	0.1 ± 0.03
M6P	4.0 ± 0.2	23.5 ± 1.1	0.2 ± 0.02	0.4 ± 0.1
2DG6P	38.4 ± 1.9	55.2 ± 1.8	0.04 ± 0.01	0.2 ± 0.02
AG6P	0.7 ± 0.1	-	-	-
R5P	8.9 ± 0.4	4.8 ± 0.3	0.2 ± 0.03	0.6 ± 0.1
E4P	6.7 ± 0.5	6.8 ± 0.6	3.8 ± 0.4	0.1 ± 0.02

G6P: glucose 6-phosphate; F6P: fructose 6-phosphate; M6P: mannose 6-phosphate; AG6P: N-acetyl-D-glucosamine-6-phosphate; 2DG6P:2-deoxy-D-glucose-6-phosphate; R5P: D-ribose-5-phosphate; E4P: D-erythrose-4-phosphate

Supplementary Table 4. Properties of enzymes and reactions in multienzyme systems

Enzymes	E.C. number	Sources	UniProt/NCBI accession No.	Sp act (U/mg)[a]	Temp (°C)	Ref.
α-Glycan phosphorylase (GP)	2.4.1.1	*Thermotoga maritima*	G4FEH8	8.9	50	1
Phosphoglucomutase (PGM)	5.4.2.2	*Thermococcus kodakarensis*	Q68BJ6	25	50	1
Glucose 6-phosphate isomerase & Mannose-6-phosphate isomerase (PGI/PMI)	5.3.1.9&5.3.1.8	*Dictyoglomus thermophilum*	B5YEP3	95	55	2
4-*α*-Glucanotransferase (4GT)	2.4.1.25	*Thermococcus litoralis*	O32462	8.9	50	3
Polyphosphate glucokinase (PPGK)	2.7.1.63	*Thermobifida fusca*	Q47NX5	65	50	4
Isoamylase (IA)	3.2.1.68	*Sulfolobus tokodaii*	Q973H3	6.4	85	5
Sucrose phosphorylase (SP)	2.4.1.7	*Bifidobacterium adolescentis*	A0ZZH6	204	60	6
Glucose 6-phosphate isomerase (PGI)	5.3.1.9	*Thermus thermophilus*	Q5SLL6	65.3	50	7

[a] Specific activity

Supplementary Table 5. Design constraints between the substrate phosphoryl group and catalytic residues of Asp8, Ser41 and Lys191.

Type	Center (Å)	Tolerance (Å)
Dis($OD2_{D8}$-O_2)	3.0	0.5
Ang($OD2_{D8}$-O2-P)	73.2	10.0
Ang(CG_{D8}-$OD2_{D8}$-O2)	120.4	10.0
Dis(OG_{S41}-O2)	2.6	0.5
Ang(OG_{S41}-O2-P)	124.2	10.0
Ang(CB_{S41}-$OD2_{S41}$-O2)	100.9	10.0
Dis(NZ_{K191}-O3)	2.6	0.5
Ang(NZ_{K191}-O2-P)	129.2	10.0
Ang(CE_{K191}-NZ_{K191}-O2)	108.4	10.0

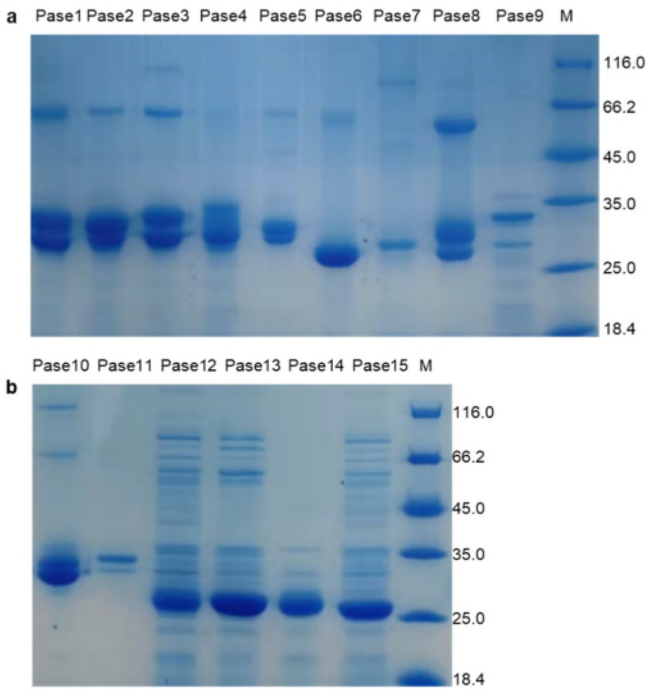

Supplementary Figure 1 SDS-PAGE analysis results of 15 candidate phosphatases in this study. **a**, Pases1-9; **b**, Pases10-15. Pases 6, 7, 8, 11 were purified by Ni^{2+} affinity chromatography, and the others were purified by heat precipitation. The SDS-PAGE analysis results were repeated for at least three times independently and one gel image is shown.

	P_G (%)	P_F (%)	P_M (%)
S126N	20.0	3.8	76.2
S126T	14.1	5.1	80.7
S126I	28.4	14.4	57.2
S126A	19.7	4.4	75.9
S126C	20.1	10.2	69.7
S126G	16.5	3.7	79.8
S126K	40.5	21.1	38.4
S126L	42.7	19.1	38.2
S126P	45.7	3.5	50.8
S126Q	19.1	10.9	70.1
S126R	23.1	8.7	68.2
S126V	17.5	4.2	78.3
H125Y	20.2	4.8	74.9
H125D	13.3	3.2	83.5
H125V	22.1	2.1	75.7
H125L	20.7	3.5	75.8
L149I	16.5	2.5	81.0
L149V	24.4	3.1	72.5
F178H	19.4	7.3	73.3
F178V	20.0	3.4	76.6
Y181F	19.4	6.7	73.8

Supplementary Figure 2 The $P_{G/F/M}$ values of single-site mutations. The reaction system contained 10 g/L maltodextrin, 10 mM of PBS (pH 6.5), 5 mM of $MgCl_2$. 0.3 mg/mL of TmGP, 0.2 mg/mL of TkPGM, 0.2 mg/mL of DtPGI/MPI, and 60 μL of heat-treated Pases and was conducted at 55°C for 4h. The P_M values were calculated by the ratio of mannose concentration to total monosaccharides concentration. The P_G values were calculated by the ratio of glucose concentration to total monosaccharides concentration. The P_F values were calculated by the ratio of fructose concentration to total monosaccharides concentration.

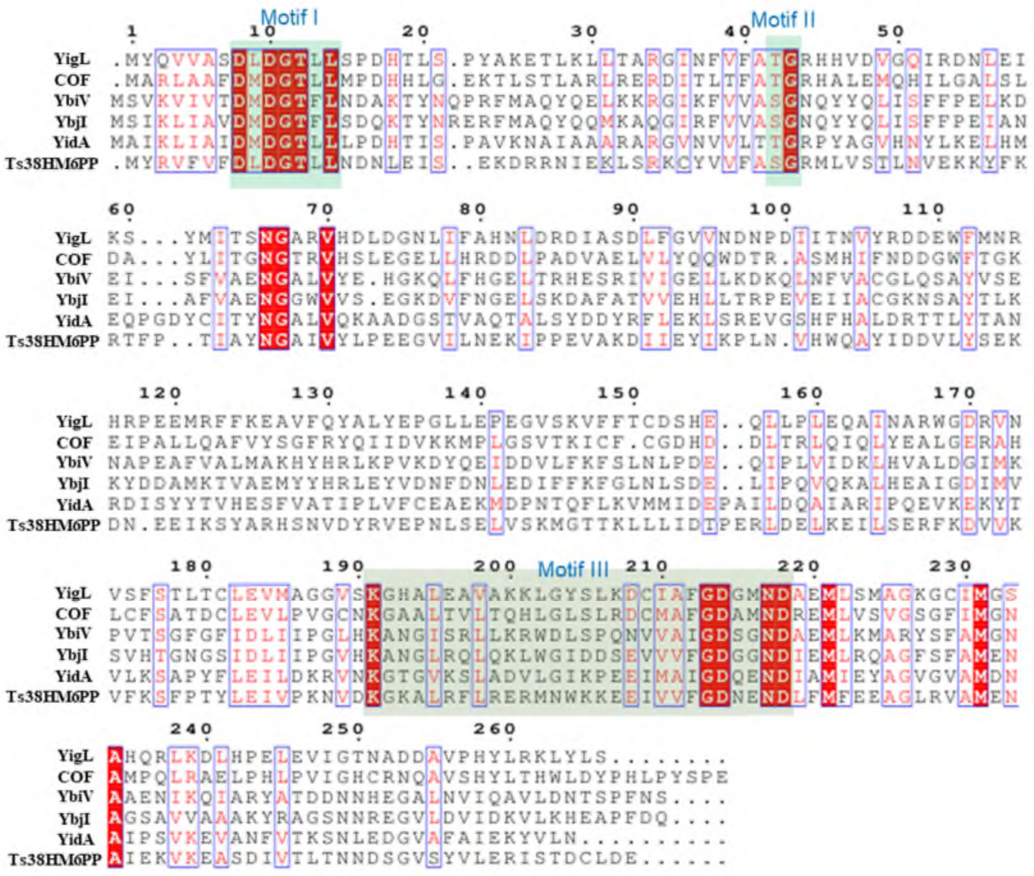

Supplementary Figure 3 Multi-sequence alignment of Ts38HM6PP with five phosphatases using ClustalW program. The protein sequences and accession numbers were presented in Supplementary data file 1.

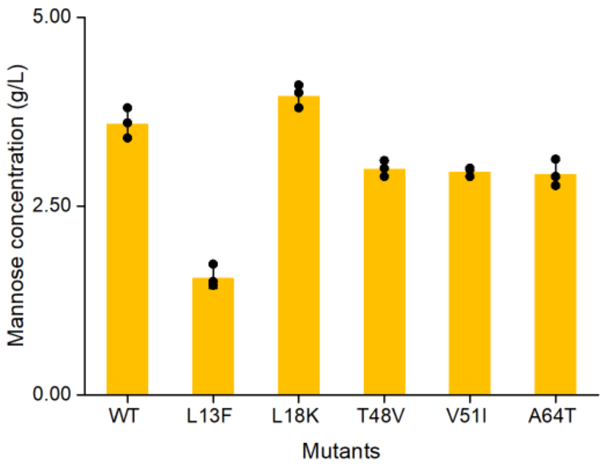

Supplementary Figure 4 The mannose concentration of different mutations in the screening system. Data are presented as mean values +/−SD (n=3 independent experiments). Source data is provided as a Source Data file.

Mutants	P_M (%)	P_F (%)	P_G (%)
L18K/T48V	87.7	1.8	10.5
L18K/V51I	88.5	1.7	9.8
L18K/A64T	87.4	1.3	11.3
T48V/V51I	82.7	1.4	16.0
T48V/A64T	84.9	0.7	14.4
V51I/A64T	80.4	1.1	18.5
L18K/V51I/A64T	86.5	2.7	10.7
S126H/Y181H	0.2	85.3	14.4
Y122E/S126D	34.9	0.8	64.2
Y122E/L9M	16.4	2.3	81.2
Y122E/L9M/E183D	19.4	2.1	78.6
Y122E/L9M/E183D/H125I	18.8	1.1	80.1
Y122E/L9M/E183D/H125I/F178L	27.7	0.5	71.8
Y122E/L9M/H125I/F178L/Y181M/L149M	8.3	0.0	91.7
Y122E/L9M/H125I/F178L/S177T/Y181M/L149M	7.4	1.6	91.0

Supplementary Figure 5 The effect of combinational mutations on $P_{G/F/M}$. The reaction system contained 10 g/L maltodextrin, 10 mM of PBS (pH 6.5), 5 mM of $MgCl_2$. 0.3 mg/mL of TmGP, 0.2 mg/mL of TkPGM, 0.2 mg/mL of DtPGI/MPI, and 60 μL of heat-treated Pases and was conducted at 55°C for 4h. The P_M values were calculated by the ratio of mannose concentration to total monosaccharides concentration. The P_G values were calculated by the ratio of glucose concentration to total monosaccharides concentration. The P_F values were calculated by the ratio of fructose concentration to total monosaccharides concentration.

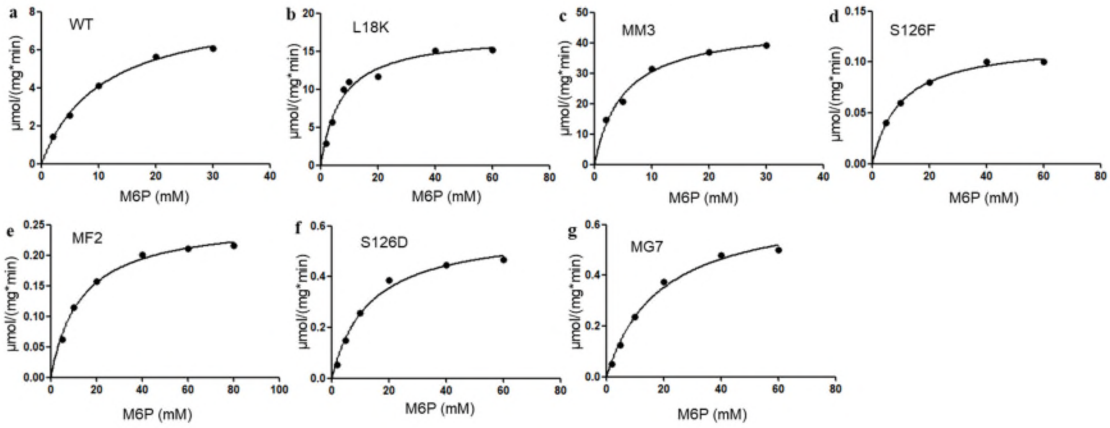

Supplementary Figure 6 The Michaelis-Menten plots for mutants of M6P. **a**, **b**, **c**, **d**, **e**, **f**, **g** represented the kinetic parameters to M6P of WT, L18K, MM3, S126F, MF2, S126D, and MG7, respectively. To measure the kinetic parameters of K_m and V_{max} of WT and mutants, the reaction mediums (200 μL) containing 10 mM of TEA buffer (pH 6.5), 5 mM of $MgCl_2$, various concentrations of M6P (2-60 mM), and purified enzymes (0.05-0.5 mg) were constructed. The reactions were performed at 55°C for 10-30 mins and stopped by adding 10% H_2SO_4 (0.5 μL).

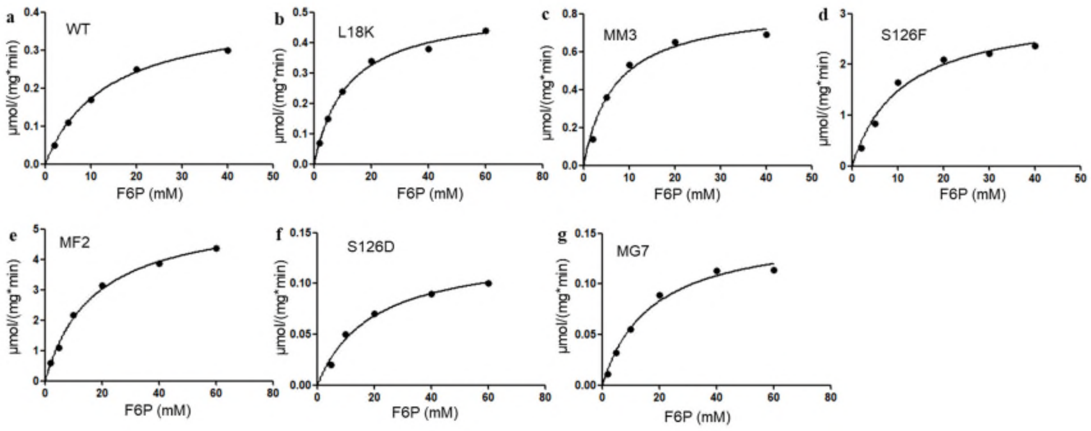

Supplementary Figure 7 The Michaelis-Menten plots for mutants of F6P. **a**, **b**, **c**, **d**, **e**, **f**, **g** represented the kinetic parameters to F6P of WT, L18K, MM3, S126F, MF2, S126D, and MG7, respectively. To measure the kinetic parameters of K_m and V_{max} of WT and mutants, the reaction mediums (200 μL) containing 10 mM of TEA buffer (pH 6.5), 5 mM of $MgCl_2$, various concentrations of F6P (2-60 mM), and purified enzymes (0.05-0.5 mg) were constructed. The reactions were performed at 55°C for 10-30 mins and stopped by adding 10% H_2SO_4 (0.5 μL).

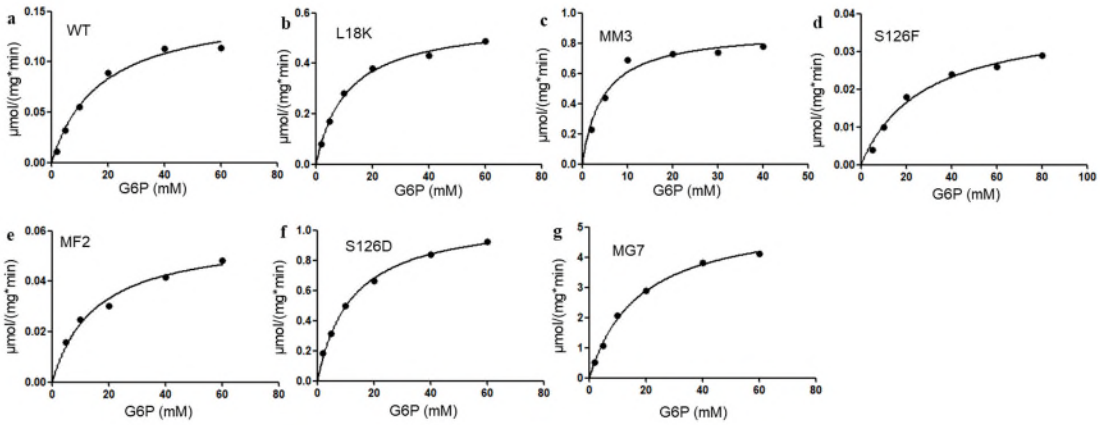

Supplementary Figure 8 The Michaelis-Menten plots for mutants of G6P. **a**, **b**, **c**, **d**, **e**, **f**, and **g** represented the kinetic parameters to G6P of WT, L18K, MM3, S126F, MF2, S126D, and MG7, respectively. To measure the kinetic parameters of K_m and V_{max} of WT and mutants, the reaction mediums (200 μL) containing 10 mM of TEA buffer (pH 6.5), 5 mM of $MgCl_2$, various concentrations of F6P (2-60 mM), and purified enzymes (0.05-0.5 mg) were constructed. The reactions were performed at 55°C for 10-30 mins and stopped by adding 10% H_2SO_4 (0.5 μL).

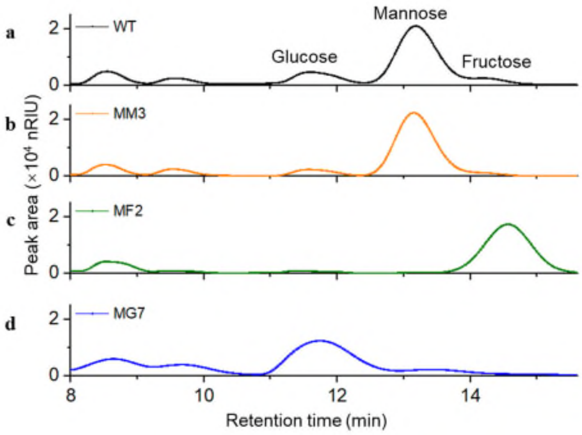

Supplementary Figure 9 HPLC chromatograms to obtain the values of $P_{M/G/F}$. **a**, **b**, **c**, and **d** represented HPLC data of $P_{M/G/F}$ values for WT, MM3, MF2, and MG7, respectively. The reaction system containing 10 g/L of maltodextrin, 10 mM of PBS (pH 6.5), 5 mM of $MgCl_2$. 0.3 mg/mL of TmGP, 0.2 mg/mL of TkPGM, 0.2 mg/mL of DtPGI/MPI, and 60 μL of enzymes was conducted at 55°C for 4h.

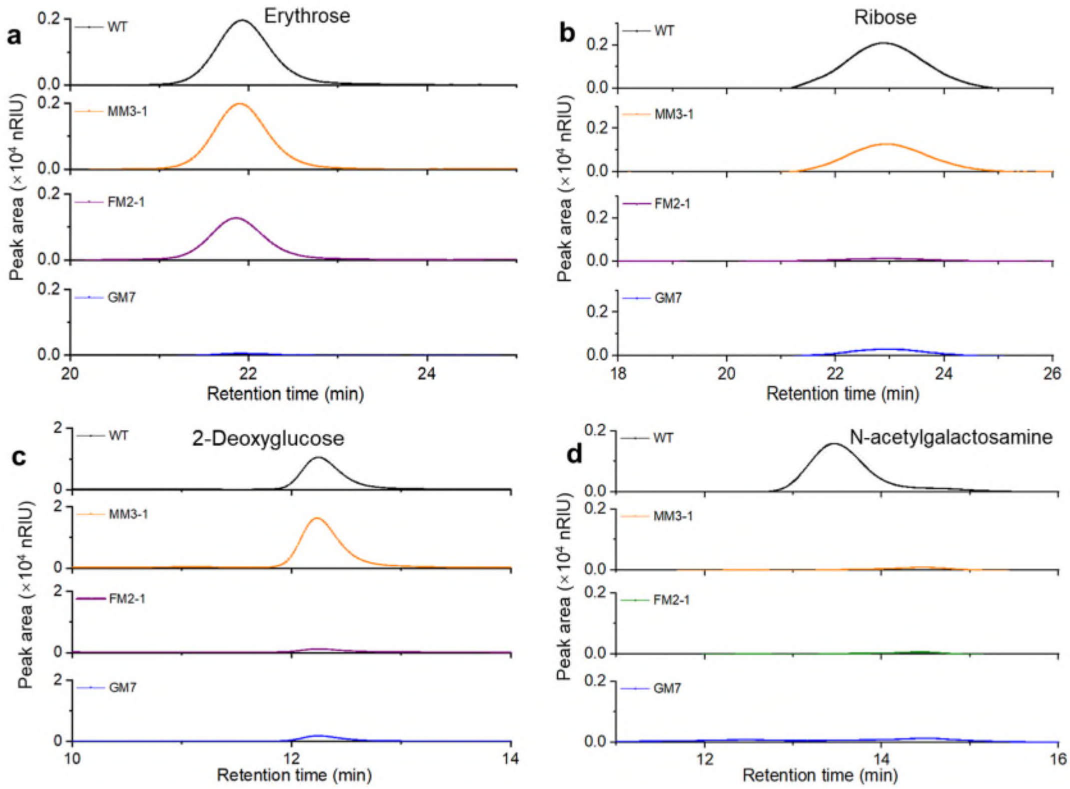

Supplementary Figure 10 The catalytic properties of WT, MM3, MF2 and MG7 to different substrates. **a**, **b**, **c**, and **d** are the HPLC chromatograms of WT, MM3, MF2 and MG7 to D-erythrose-4-phosphate (E4P), D-ribose-5-phosphate (R5P), 2-deoxy-D-glucose-6-phosphate (2DG6P), and N-acetyl-D-glucosamine-6-phosphate (AG6P), respectively.

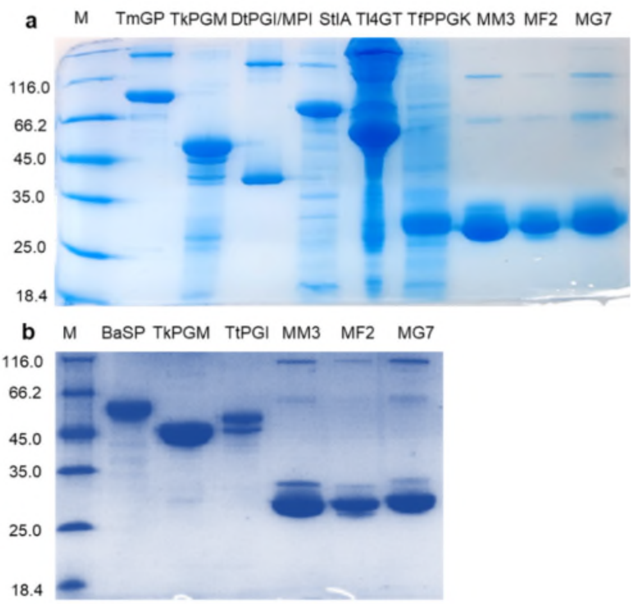

Supplementary Figure 11 SDS-PAGE analysis of enzymes used in the biosynthetic systems. **a**, The SDS-PAGE analysis results of enzymes for fructose and mannose production from maltodextrin. **b**, The SDS-PAGE analysis results of enzymes for fructose and mannose production from sucrose. The SDS-PAGE analysis experiments were repeated for at least three times independently and one gel image is shown.

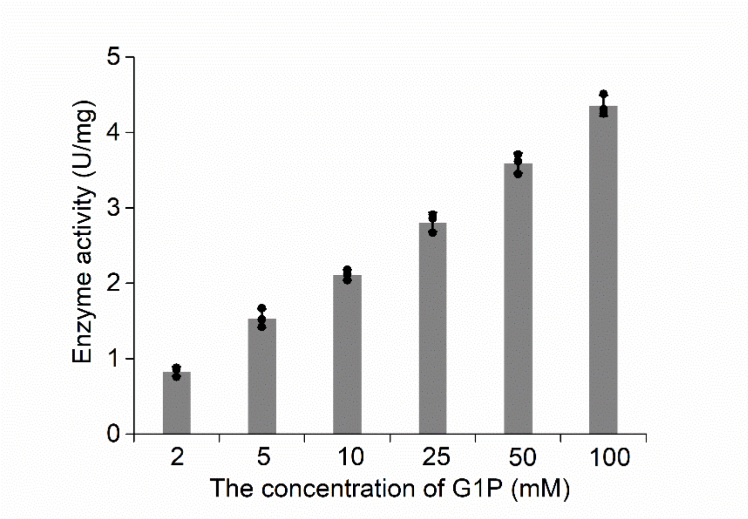

Supplementary Figure 12 The dephosphorylation activity of BaSP to G1P. Data are presented as mean values +/−SD (n=3 independent experiments). Source data is provided as a Source Data file.

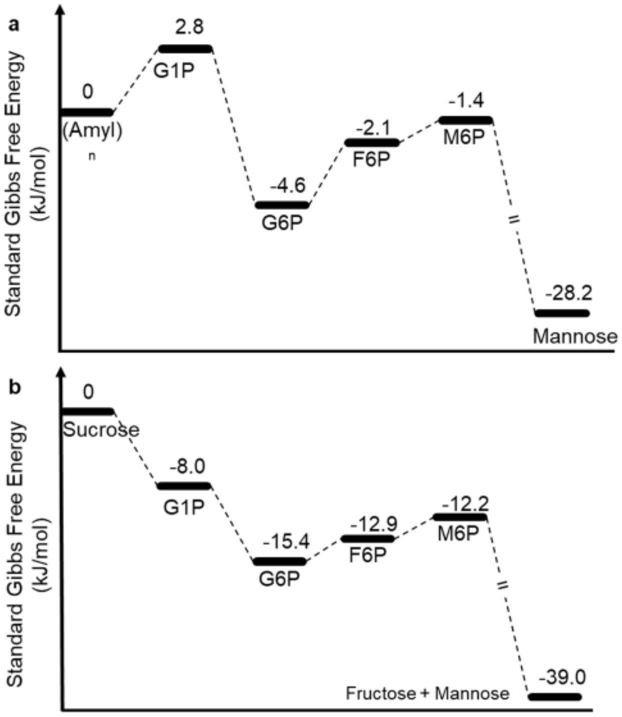

Supplementary Figure 13 Standard Gibbs free energy change of each and overall reaction for manufacturing mannose in the biosystem. **a**, the standard Gibbs free energy change for mannose production from maltodextrin. **b**, the standard Gibbs free energy change for mannose production from sucrose. The ΔG° represents the change of Gibbs free energy and was freely available on the website. http://equilibrator.weizmann.ac.il.

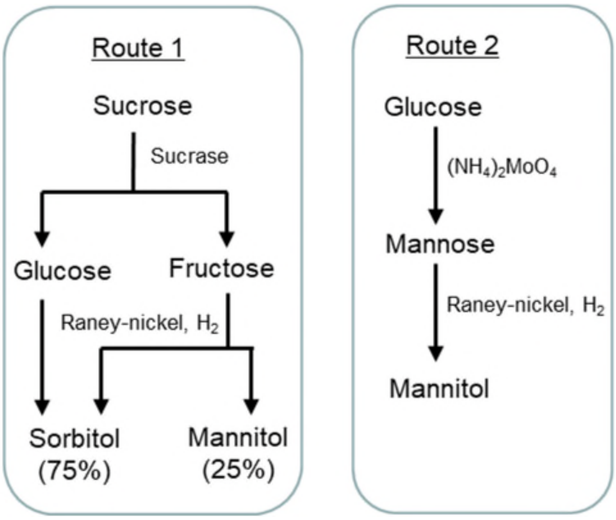

Supplementary Figure 14 Two industrial routes for manufacturing mannitol. The routes 1 and 2 were mentioned in previous studies[8,9].

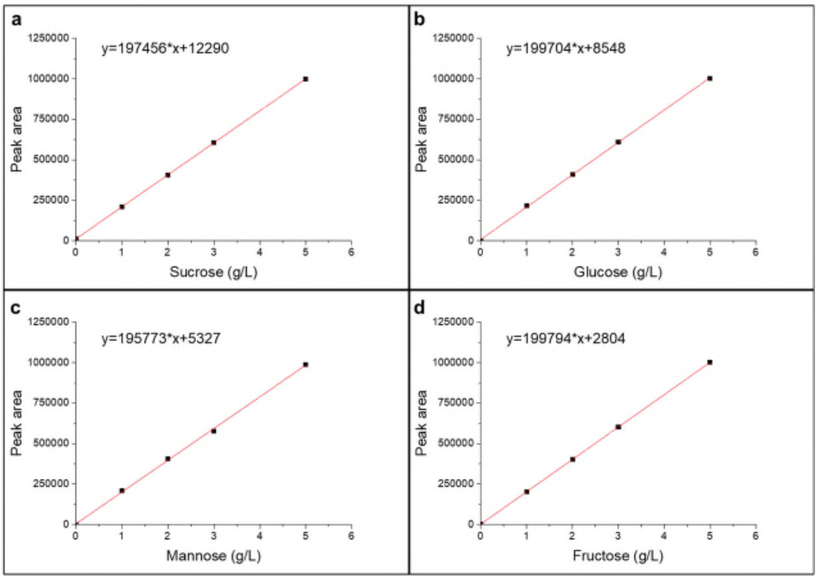

Supplementary Figure 15 The calibration curve for sugars. **a**, **b**, **c**, and **d** represent the HPLC calibration curves for sugars sucrose, glucose, mannose, and fructose, respectively.

Supplementary References

[1] You, C., Shi, T., Li, Y., Han, P., Zhou, X., & Zhang, Y.-H. P. An *in vitro* synthetic biology platform for the industrial biomanufacturing of *myo*-inositol from starch. *Biotechnol. Bioeng.* **114**, 1855-1864 (2017).

[2] Tian, C., Yang, Y., Li, Y., Zhang, T., Li, J., Ren, C., Men, Y., Chen, P., You, C., Sun, Y., & Ma, Y. Artificially designed routes for the conversion of starch to value-added mannosyl compounds through coupling *in vitro* and *in vivo* metabolic engineering strategies. *Metab. Eng*. **61**, 215-224 (2020).

[3] Jeon, B. S., Taguchi， H., Sakai， H., Ohshima， T., Wakagi， T., Matsuzawa, H. 4-α-Glucanotransferase from the hyperthermophilic archaeon *Thermococcus litoralis. FEBS J.* **248**, 171-178 (2010).

[4] Zhou, W., Huang, R., Zhu, Z., & Zhang, Y.-H. P. J. Coevolution of both thermostability and activity of polyphosphate glucokinase from *Thermobifida fusca* YX. *Appl. Environ. Microbiol*. **84**, e01224-18 (2018).

[5] Cheng, K., Zhang, F., Sun, F., Chen, H., & l Zhang, Y. P. H. Doubling power output of starch biobattery treated by the most thermostable isoamylase from an archaeon *Sulfolobus tokodaii*. *Sci. Rep*. **5**, 13184 (2015).

[6] Cerdobbel, A., Winter, K. D., Aerts, D., Kuipers, R., Joosten, H., Soetaert, W. & Desmet, T. Increasing the thermostability of sucrose phosphorylase by a combination of sequence- and structure-based mutagenesis. *Protein Eng., Des. Sel*. **24**, 829-834 (2011).

[7] Li, Y., Shi, T., Han, P., & You, C. Thermodynamics-driven production of value-added D-allulose from inexpensive starch by an in vitro enzymatic synthetic biosystem. *ACS Catal.* **11**, 5088-5099 (2021).

[8] Motohiro, T. S. & Mochihiro, I. K. Process for preparing d-mannitol. US4083881 (1976).

[9] Francis, D. Process for the manufacture of mannitol. US5466795 (1995).
