# Supplementary material for: Engineering substrate specificity of HAD phosphatases and multienzyme systems development for the thermodynamic-driven manufacturing sugars
Source: Nat Commun. 2022 Jun 23;13:3582. doi: 10.1038/s41467-022-31371-8 (PMC9226320; doi:10.1038/s41467-022-31371-8)
Supplement: Supplementary file 3 — Description of Additional Supplementary Files [file 41467_2022_31371_MOESM3_ESM.pdf]

Description of Additional Supplementary Files

File Name: Supplementary Data 1
Description: The protein sequence of 15 candidate phosphatases and five phosphatases from *E.coli*

File Name: Supplementary Data 2
Description: Primers used in this study.

File Name: Supplementary Data 3
Description: Protein expression plasmids
